# Balloon expandable transcatheter aortic valve implantation via the transfemoral route with or without pre-dilation of the aortic valve – rationale and design of a multicentre registry (EASE-IT TF)

**DOI:** 10.1186/s12872-016-0390-4

**Published:** 2016-11-15

**Authors:** Christian Butter, Peter Bramlage, Tanja Rudolph, Claudius Jacobshagen, Jürgen Rothe, Hendrik Treede, Sebastian Kerber, Derk Frank, Lenka Seilerova, Gerhard Schymik

**Affiliations:** 1 Department of Cardiology, Immanuel Clinic Bernau, Heart Center Brandenburg, Ladeburger Straße 17, 16321 Bernau, Germany; 2 Institute for Pharmacology and Preventive Medicine, Cloppenburg, Germany; 3 Herzzentrum, Universitätsklinikum Köln, Cologne, Germany; 4 Herzzentrum, Universitätsklinikum Göttingen, Göttingen, Germany; 5 Division of Cardiology and Angiology II, University Heart Center Freiburg-Bad Krozingen, Bad Krozingen, Germany; 6 Universitätsklinik und Poliklinik für Herzchirurgie, Universitätsklinikum Halle, Halle, Germany; 7 Herz- und Gefäß-Klinik, Bad Neustadt, Germany; 8 ZHK (German Centre for Cardiovascular Research), Partner Site Kiel/Hamburg/Lübeck, Hamburg, Germany; 9 Department of Internal Medicine III (Cardiology and Angiology), University Hospital Schleswig-Holstein, Campus Kiel, Germany; 10 Edwards Lifesciences, Prague, Czech Republic; 11 Medical Clinic IV, Department of Cardiology, Municipal Hospital, Karlsruhe, Germany

**Keywords:** Transcatheter aortic valve implantation, Transfemoral, EASE-IT, Balloon aortic valvuloplasty (BAV), Direct TAVI, Edwards SAPIEN

## Abstract

**Background:**

Transcatheter aortic valve implantation via the transfemoral route (TF-TAVI) is commonly performed as a treatment for severe aortic stenosis (AS) in patients at high surgical risk. Pre-deployment balloon aortic valvuloplasty (BAV) has generally been considered an essential step for preparing the valve landing zone for receipt of the prosthesis. However, there is little evidence supporting the clinical value of BAV, while several associated complications have been documented. This has provoked several groups to evaluate the feasibility and safety of omitting BAV form the TF-TAVI procedure (direct TF-TAVI), with encouraging results. However, studies comparing the clinical outcomes of direct TF-TAVI to standard TF-TAVI are lacking.

**Methods:**

EASE-IT TF is a prospective, observational, two-armed, multicentre registry designed to gather data on procedural aspects, adverse events and survival rates associated with direct TF-TAVI using the Edwards SAPIEN 3 balloon-expandable prosthesis.

**Discussion:**

EASE-IT-TF data will be analysed firstly to determine the risks and benefits associated with direct TF-TAVI vs. standard TF-TAVI, and secondly to identify associations between patient variables and specific outcomes. This may assist identification of patients who stand to benefit from direct TF-TAVI, therefore contributing to clinical reductions in TF-TAVI-associated morbidity and mortality rates in high-risk AS patients.

**Trial registrations:**

Clinictrials.gov: NCT02760771

## Background

Until recently, pre-dilation of the stenosed aortic valve using an expandable balloon (pre-deployment balloon aortic valvuloplasty; BAV) has been deemed an essential step for facilitating the crossing of the aortic annulus, reducing radial counterforce, and permitting greater prosthetic heart valve (PHV) expansion during transcatheter aortic valve implantation (TAVI) [1], though there is little proof of this in the literature. BAV may also permit early evaluation of cusp positioning, calcification, and the potential for balloon slippage during the successive valve landing [2]. Conversely, there are several problems associated with BAV, including transient coronary, cerebral, and renal ischemia, haemodynamic instability and systemic inflammatory response syndrome as a result of the need for rapid ventricular pacing (>180 bpm for up to 30 s) [2]; conduction disturbances requiring permanent pacemaker implantation (PPI) [3, 4]; coronary occlusion; tamponade; profound hypotension; aortic regurgitation; and displacement of fragments from the valve increasing stroke risk [4–7]. With this in mind, modification of the TAVI procedure to omit the BAV step has been investigated in several clinical studies for a variety of access routes, and generally demonstrated to be feasible and safe without compromising procedural success [1, 5, 8–14].

Several pilot studies in high surgical risk patients undergoing TF-TAVI without prior BAV (direct TF-TAVI) have been published to date. An early pilot study by Grube et al. in 60 consecutive patients undergoing direct TF-TAVI with the Medtronic CoreValve PHV described a 96.7 % procedural success rate, with in-hospital mortality, stroke, major vascular complication, and myocardial infarction (MI) rates of 6.7, 5, 10, and 0 %, respectively [1]. Concurrently, a similar pilot study by Mendiz et al. using the same PHV demonstrated that direct TF-TAVI in 51 patients led to a procedural success rate of 94.2 %, with PPI, stroke and all-cause mortality rates of in 27.5, 3.9, and 3.9 % at 30 days [11]. Most recently, a larger retrospective study enrolling 163 patients found that direct TF-TAVI with the SAPIEN 3 PHV was feasible in 94.5 % of cases, and had an all-cause mortality rate of 3.7 % at 30 days [10]. BAV was required to overcome crossing difficulties in only 5.5 % of patients, who were generally older, had a higher calcium score, higher transvalvular gradients, and smaller AVA. Thus, pilot studies have demonstrated the feasibility of direct TF-TAVI in the majority of standard TF-TAVI candidates; though suggest that further studies to identify characteristics of patients for whom the technique is most appropriate would be useful.

Early, small comparative studies found that compared to standard TF-TAVI, direct TF-TAVI resulted in higher success rates (85 % vs. 64 %), a significantly lower incidence of moderate-to-severe paravalvular leak (9 % vs. 33 %) [12], reduced radiation dosage (42.0 vs. 56.6 Gy cm^2^), and reduced contrast agent volume (92.2 vs. 112 mL) [13]. Bijkulic et al. found, guided by diffusion-weighted magnetic resonance imaging, that the implantation of a balloon-expandable aortic valve without versus with prior BAV, although performed with a shorter procedure time and lower contrast volume, is associated with a significantly higher volume of cerebral ischemic lesions [15]. A later, case-matched analysis in 52 patients using Edwards SAPIEN PHVs found no significant difference between direct TF-TAVI and standard TF-TAVI in terms of success rate (96.2 % vs. 92.3 %), all-cause mortality at 30 days (both 7.7 %), disabling stroke (3.8 % vs. 7.7 %) or moderate-severe paravalvular leak (0 % vs. 7.7 %); though did document significantly lower procedural times (60 vs. 70 min), fluoroscopy time (13.3 vs. 17.8 min), and contrast agent volume (118.7 vs. 153.0 mL) for direct TF-TAVI [8]. However, the insufficient statistical power and unsatisfactory study design of the aforementioned studies prevent firm conclusions being drawn. One larger study by Islas et al. in 249 consecutive patients undergoing TF-TAVI was published recently, and found that direct TAVI resulted in significantly shorter procedural durations (108.5 vs. 133.7 min), reduced rates of PPI (6.3 % vs. 14.1 %), procedure-related mortality, and 30-day mortality (both 2.5 % vs. 11.8 %) [16]. While these results are encouraging, it should be noted that group assignment was based on pre-defined transesophageal echocardiographic criteria potentially biasing patient populations, the study was monocentric, and employed two different PHVs (only 106 patients received the Edwards SAPIEN PHV and 143 patients the CoreValve). Thus, findings should be interpreted with care. Considered together, early comparative studies suggest that direct TF-TAVI may offer several advantages over standard TF-TAVI, though carefully designed, multi-centric, large-cohort studies are clearly required.

To the best of our knowledge, no large registry, fully-prospective studies comparing direct TF-TAVI to standard TF-TAVI with the Edwards SAPIEN PHVs have been published to date. We have therefore designed a two-armed, controlled-cohort, observational, multicentre registry (EASE-IT TF) to investigate the clinical implications of BAV in TF-TAVI using the balloon expandable Edwards SAPIEN 3 PHV. Data on procedural aspects, adverse events and mortality will be used to identify differences in operative outcomes when BAV is performed or omitted. Possible associations between patient variables and these outcomes will then be identified.

## Methods/design

This prospective, non-randomised, non-interventional controlled cohort study evaluating the safety and efficacy of the Edwards SAPIEN 3 PHV in standard TF-TAVI vs. direct TF-TAVI is based on EASE-IT TF: a two-armed, observational registry containing data from 10 German sites. Ethic committee approval was obtained from the appropriate ethics committee in writing prior to patient an enrolment. We aim to enrol and average of 20 patients per site, generating a minimum study population of 200. All patients are required to provide a signed informed consent form (ICF) prior to enrolment.

### Site selection

Selected sites must have prior experience of TF-TAVI implantations and the proven capacity to enrol at least 2 patients per month. Additionally, independently of inclusion in the registry, all participating sites must have received the following training for implantation of the Edwards SAPIEN 3: 1) instructions for use (IFU) provided by the manufacturer, 2) exhaustive fundamentals training (i.e., device preparation, didactic sessions, simulator training and case observations), and 3) on-site training as per the Edwards Standard Operating Procedure.

### Patient selection

**Fig. 1:**
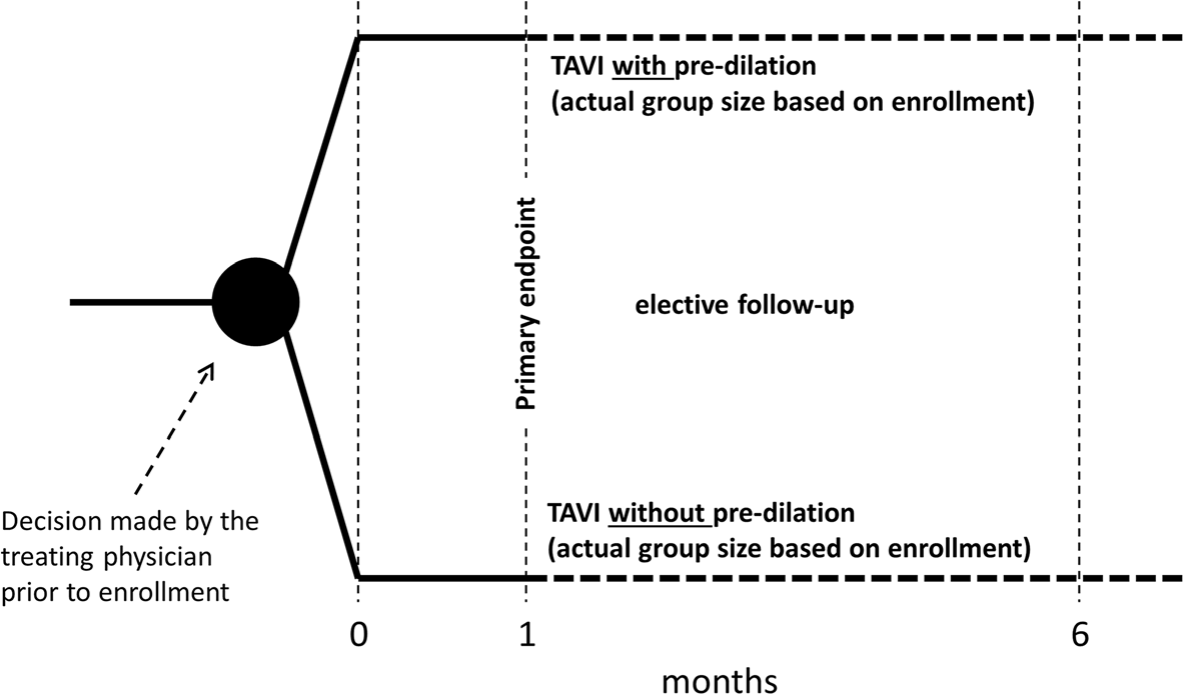
Diagram of registry design, procedures and stages

In order to be included in the study, patients are required to meet all of the following criteria: 1) indication for TF-TAVI according to the IFU for the Edwards SAPIEN 3 PHV, 2) age ≥18 years, 3) signed ICF. All patients meeting any of the following criteria will be excluded from the study: 1) contraindications for TAVI via the TF access route according to the Edwards SAPIEN 3 IFU, 2) logistic Euro-SCORE I >40 %, 3) mitral or tricuspid valvular insufficiency > grade II, 4) uncontrolled atrial fibrillation (no rhythm control established), 5) left ventricular or atrial thrombus identified via echocardiography, 6) previous aortic valve replacement, 7) mobile structures on leaflets, 8) requirement for a cerebral protection device, and 9) high probability of non-adherence to follow-up requirements based on social, psychological or medical grounds. So as to avoid influencing investigator decision to perform TF-TAVI with or without BAV, enrolment in the register will occur after this choice had already been made, and a 1:1 ratio will not be strictly pursued (Fig. 1). Reasons for this decision and any subsequent changes will be systematically documented. Subjects withdrawing consent will be omitted from the registry and not replaced.

### TF-TAVI procedure

Investigators are free to perform TF-TAVI using the Edwards SAPIEN 3 PHV according to locally-adopted techniques. Patients in the standard TF-TAVI group will undergo the procedure with pre-deployment BAV, while those in the direct TF-TAVI group will undergo the procedure without pre-deployment BAV.

### Outcome measures

The primary outcome measure is the combined rate of all-cause mortality, stroke, non-fatal MI, acute kidney injury (AKI), and PPI between commencement of the TAVI procedure and 30 days (±7 days) post-intervention. Secondary outcome measures are each of the aforementioned factors making up the primary outcome considered separately at 30 days (±7 days) and 6 months (±16 days) post-TAVI, procedure time, duration of radiation exposure, volume of contrast agent used, and rate of aortic root rupture. A further secondary outcome measure is the combined rate of all-cause mortality, stroke, non-fatal MI, AKI, and PPI up to 6 months (±14 days) post-TAVI.

### Data collection

Data on enrolled patients will be collected via electronic case report forms (eCRFs) using registry identification numbers rather than names, to protect identity. eCRFs will be completed for the visit prior to implantation, on the day of implantation, and at follow-up visits 30 ± 7 days and 6 months (±14 days) post-implantation, and signed at the earliest opportunity by either the investigator or designated responsible party (Table 1). The results of pre-TAVI echo and CT assessment will be documented as well as potential reasons for or against opting for BAV in a given patient. Additionally, reports of device-related serious adverse events or death must be provided within 48 h of occurrence. Up to 2 sites (20 %) will be randomly selected for monitoring purposes following completion of data collection, and subjected to source data verification (expected to be 100 % for stroke, life-threatening or major bleeding, renal failure, vascular and valve complications, PPI and mortality).

**Table 1:** Assessments

Parameter	Admission	Intervention	Discharge	30d FU	6 Mo FU
Inclusion/Exclusion criteria	X				
Demographics	X				
Diagnosis of valve disease	X				
Echocardiography	X				
Computed Tomography	X				
Symptoms	X			X	X
Cardiac baseline characteristics	X				
ECG	X		X		
Comorbidities	X				
Risk scores	X				
Prior cardiovascular intervention	X				
Current medications	X		X	X	X
Interventional details		X			
Interventional results		X			
AE		X	X	X	X
Hospitalization duration			X		
Creatinine value	X		X		X
Early safety/Clinical efficacy				X	X

### Statistics

To achieve a statistical power of 80 %, the estimated sample size needed to detect an absolute risk reduction of 13 % at 30 days post-TAVI was 180 evaluable patients. This calculation was based on the 30-day event rates reported by a previous study on direct TF-TAVI by Grube et al. [1]. In order to accommodate an approximate drop out rate of 10 % (20 patients) during the first 30 days, a final necessary sample size of 200 patients was established.

Analysis for all patients in the registry will be performed from an intention-to-treat perspective. The collected data will be presented and summarised using descriptive statistics. Categorical variables will be reported as frequency distributions, while continuous variables will be reported as mean (± standard deviation) and median (range) values. Where appropriate, actuarial probability and linearised rates may be used to report on adverse events. Survival (and adverse event outcomes, where appropriate) will be analysed using the Kaplan-Meier method.

## Discussion

The present registry-based controlled cohort study (EASE-IT TF) aims to assess the procedural and clinical outcomes of TF-TAVI with the Edwards SAPIEN 3 PHV when BAV is performed, compared to when the BAV step is omitted. Analysis of the resulting data should provide insight into the clinical relevance and value of BAV in TF-TAVI, and also reveal possible patient variables associated with different outcomes. These findings will have several notable implications for clinical practise.

Despite a number of groups reporting the feasibility and safety of direct TF-TAVI with the Edwards SAPIEN PHV [8, 10, 13, 16], the relative impact of this technique on clinical outcomes has not been properly verified due to methodological limitations of existing comparative studies. As a well-designed, two-arm, fully-prospective, real-world registry, EASE-IT TF will shed light on the potential for direct TF-TAVI to reduce the incidence of the most eminent serious adverse events currently facing TF-TAVI patients. Most notably, these include stroke, AKI, MI, conduction disturbances requiring permanent PPI, and mortality.

Stroke has been previously identified as a predictor of early and medium-term mortality in TAVI patients [17]. It is thought that stroke during TAVI occurs due to dislodgement of embolic fragments from the valve and access vessels caused by the extensive movement of catheters and guide wires, BAV, and PHV expansion. In a study by Reinsfelt et al. using intraoperative transcranial Doppler imaging, 282 cerebral microembolic signals (MES) were recorded in patients undergoing routine TAVI, of which 37 % were generated during instrumentation and 22 % during BAV [18]. A similar Doppler-based study by Erdoes et al. detected a median of 580 intraoperative cerebral high-intensity transient signals (HITS) in TF-TAVI patients, with 28 % generated during instrumentation and 11 % during BAV [7]. A further study by Drews et al. detected medians of 435 and 471 HITS and 78 and 62 MES in the right and left middle cerebral arteries of TAVI patients, respectively, with particular peaks during BAV and PHV positioning [6]. These studies suggest that elimination of BAV from the TAVI procedure (thereby also reducing instrument manipulation) may reduce stroke risk, and potentially minimise other thromboembolic outcomes such as MI. EASE-IT TF will be valuable for testing this hypothesis.

A frequently observed clinical complication of TAVI is AKI, which has been documented in around 16 % of patients and is associated with poor prognosis [19–22]. One factor that may contribute to AKI is the volume of contrast agent used. Van Linden et al. reported a significant correlation between intraoperative contrast agent volume and postoperative AKI in 270 consecutive patients undergoing TA-TAVI with the Edwards SAPIEN PHV [22]. In particular, use of >99 ml of contrast agent was an independent predictor of AKI. Concurrently, a study by Yamamoto et al. in a cohort of 415 elderly TF-TAVI patients found that subjects experiencing post-procedural AKI had been exposed to greater contrast agent doses, and multivariate analysis confirmed the association [21]. Furthermore, a study by Madershahian et al. including 50 patients with pre-existing renal impairment undergoing TA-TAVI with the Edwards SAPIEN PHV found a significantly greater prevalence of acute contrast-induced nephropathy in patients receiving >100 mL contrast agent compared to those receiving <100 mL (69.2 % vs. 41.7 %, respectively) [23]. It therefore appears that reducing contrast agent volume may reduce AKI rates. Importantly, several studies report that direct TAVI significantly reduces contrast agent volumes [8, 13], indicating the potential for this to translate into reduced AKI. Again, results from the EASE-IT TF registry will help to clarify this idea.

Conduction disturbances requiring PPI have been reported to occur in 13–25 % of patients undergoing TAVI [3, 24]. In PARTNER, patients requiring PPI had longer postoperative hospitalisation times (7.3 days vs. 6.2 days), higher frequencies of repeat hospitalisations up to 1 year (23.9 % vs. 18.2 %) and increased mortality or repeat hospitalisation up to 1 year (42.0 % vs. 32.6 %) [25]. Therefore, reducing PPI requirement is clearly desirable. In a study by Gensas et al., BAV was identified as an independent predictor of PPI requirement [3]. Concurrently, findings by Grube et al., Fiorina et al. and Islas et al. suggest that PPI rate is lower in patients undergoing direct TF-TAVI compared to standard TF-TAVI (11.7 % vs. 27.8 %, 5.5 % vs. 15.5 %, and 6.3 % vs. 14.1 %, respectively). Conversely, Conradi et al. found no significant difference in PPI rate between the two procedural variants (both 15.4 %). Therefore, the effect of BAV on PPI requirement requires additional investigation, which the EASE-IT TF study will provide.

In addition to identifying potential benefits, the EASE-IT TF study may also reveal negative outcomes associated with direct TF-TAVI. These may include the need for post-procedural BAV, which has already been noted in 16.7–34 % of patients undergoing direct TF-TAVI with the CoreValve PHV [1, 11, 12] and 0–13.7 % of patients with an Edwards SAPIEN PHV [8, 13, 16]. Data gathered in EASE-IT TF will provide additional comparative evidence on this, and other complications of direct TF-TAVI.

Taking everything into account, EASE-IT TF should provide invaluable information from which to make well-informed recommendations about the costs/benefits of performing BAV in TF-TAVI. The planned analysis to identify specific patient characteristics associated with more or less favourable outcomes (for example, exclusion of BAV is associated with reduced AKI rate in patients with existing kidney disease, while detrimental in patients with reduced left ventricular ejection fraction) has the potential to offer valuable insight into the appropriateness of BAV in distinct patient subsets. In terms of future developments within the field, EASE-IT TF will promote critical consideration of the currently adopted TAVI method, and may lead to refinement of technical guidelines.

### Potential limitations of EAE-IT TF

As an observational study, EASE-IT TF is extremely useful for providing real-world information that is directly applicable to clinical practise, but has several potential limitations. Firstly, physicians are free to place patients in either direct TF-TAVI or standard TF-TAVI groups at their discretion, which may mean more patients with a certain characteristic (i.e., higher Euro-SCORE) are concentrated in a particular group. This lack of randomisation may produce distinct study arm populations, limiting validity of subsequent inter-group comparisons. However, this is unavoidable if we wish to assess the benefits of direct TF-TAVI in patients that physicians consider eligible in the real world. By asking investigators to justify their decision, we gain valuable information about the criteria currently deemed indicative for TF-TAVI with or without BAV. This will enable us to reflect upon the reliability of these factors for determining direct TF-TAVI eligibility, and provide further recommendations. Secondly, the fact that a 1:1 group assignment ratio is not obligatory may result in smaller patient numbers in one study arm, potentially reducing statistical power. Again, this is an inherent and unavoidable problem when examining real-world circumstances. Thirdly, compared to most clinical trials, observational registry data tends to contain more omissions and inaccuracies. However, monitoring following database closure is planned, and will ensure verification of source data in a reasonable sample of enrolled patients to minimise this limitation. Finally, only a 6-month follow-up is planned, which may be inadequate to detect the effect of direct TF-TAVI on long-term outcomes. Further studies may therefore be required to evaluate these long-term consequences. Though essential for validity, the use of a single PHV type (Edward SAPIEN 3) in EASE-IT TF means that findings will be limited to TF-TAVI with this specific device. Therefore, further studies assessing the clinical value of BAV using other PHVs will be necessary. The same is true for alternative delivery systems, access routes and patient groups.

## Conclusions

The large-cohort EASE-IT-TF study will generate important data regarding rates of procedural success, adverse events, and mortality associated with omitting BAV from the standard TF-TAVI procedure. This information will be used to determine the risks and benefits associated with direct TAVI, which may then be applied to clinical practise for the identification of appropriate patients. In this way, EASE-IT TF will contribute to reductions in morbidity and mortality rates associated with TAVI in high-risk AS patients.
